# An improved leaping detector for flow analysis applied to iron speciation in drugs

**DOI:** 10.1155/S1463924600000092

**Published:** 2000

**Authors:** Sérgio R. B. Santos, Mário C. U. Araújo, Ricardo S. Honorato, Elias A. G. Zagatto, José F. C. Lima, Rui A. S. Lapa

**Affiliations:** 1 Departamento de Química Universidade Federal da Paraíba P.O. Box 5093 João Pessoa PB 58051-970 Brazil; 2 Centro de Energ ia Nuclear na Agricultura Universidade de São Paulo Piracicaba SP Brazil; 3 Faculdade de Farmácia Universidade do Porto Portugal

## Abstract

A low inner volume (ca. 64 ml) probe was built up in an injector-commutator in order to behave as a photometric leaping detector in flow analysis. It comprises a bicolour light-emitting diode (BLED), as a source of pulsed radiation in the red and green visible region, and two phototransistors as transducers. Sample injection, detector relocation, analytical signal recording, data treatment and definition of the spectral working range were computer-controlled. The feasibility of the system was initially demonstrated in the flow-injection speciation of iron, and the overall standard deviation of results was estimated as ± 1.6 and ± 1.4% for 1.6–4.0 mg l^−1^ Fe(II) or total iron after eightfold processing of synthetic samples. The system was further applied to drug analysis: the mean deviations of results for typical samples were estimated as ± 5.2 and ± 3.3%, and the relative standard deviation as ± 1.6 and ± 1.3% for Fe(II) and total iron, respectively. Results were compared with those obtained by a conventional spectrophotometric procedure and no statistic differences at the 95% confidence level were found. In relation to an earlier system with multi-site detection, the proposed system is more stable, presenting low drift with a relative standard deviation of 0.026% and 0.039% for measurements (n=120 during 4 h of observation) with green and red emission. It is also faster with a sampling rate of 133 h^−1^ and carryover problems are not found. The possibility of compensating the Schlieren noise by dual-wavelength spectrophotometry is discussed.


Journal of Automated Methods & Management in Chemistry, Vol. 22, No. 3 (May–June 2000) pp. 83–88

### An improved leaping detector for flow analysis applied to iron speciation in drugs

**Sérgio R. B. Santos*, Mário C. U. Araújo, Ricardo S. Honorato, Elias A. G. Zagatto,[1] José F. C. Lima[2] and Rui A. S. Lapa[2]**

*Departamento de Química, Universidade Federal da Paraíba, P.O. Box 5093, João Pessoa PB 58051-970, Brazil, [1] Present address: Centro de Energia Nuclear na Agricultura, Universidade de São Paulo, Piracicaba SP, Brazil, [2] Present address: Faculdade de Farmácia, Universidade do Porto, Portugal*

*A low inner volume (ca. 64 ml) probe was built up in an injector-commutator in order to behave as a photometric leaping detector in flow analysis. It comprises a bicolour light-emitting diode (BLED), as a source of pulsed radiation in the red and green visible region, and two phototransistors as transducers. Sample injection, detector relocation, analytical signal recording, data treatment and definition of the spectral working range were computer-controlled. The feasibility of the system was initially demonstrated in the flow-injection speciation of iron, and the overall standard deviation of results was estimated as ±1.6 and ±1.4% for 1.6–4.0 mg $l^{-1}$ Fe(II) or total iron after eightfold processing of synthetic samples. The system was further applied to drug analysis: the mean deviations of results for typical samples were estimated as ±5.2 and ±3.3%, and the relative standard deviation as ±1.6 and ±1.3% for Fe(II) and total iron, respectively. Results were compared with those obtained by a conventional spectrophotometric procedure and no statistic differences at the 95% confidence level were found. In relation to an earlier system with multi-site detection, the proposed system is more stable, presenting low drift with a relative standard deviation of 0.026% and 0.039% for measurements (n = 120 during 4 h of observation) with green and red emission. It is also faster with a sampling rate of 133 $h^{-1}$ and carryover problems are not found. The possibility of compensating the Schlieren noise by dual-wavelength spectrophotometry is discussed.*

### Introduction

In flow spectrophotometry, multiple measurements on a processed sample are efficiently accomplished in order to permit implementation of simultaneous determinations, differential kinetic analysis, speciation, standard additions, titrations, blank compensation, etc. For this task, a diode array spectrophotometer [1], or a single instrument in association with different strategies, e.g. stream splitting [2], closed-loop configuration [3], sandwich techniques [4, 5], flow reversal [6], oscillating streams [7], parallel streams towards the instrument optical beam [1], etc. have been used. Alternatively, several detectors can be placed at specific sites of the manifold [8, 9]. In the above-mentioned examples, however, the detectors act in a motionless way and, as a consequence, the sampling rate is usually dependent on the time interval required for passage of the entire sample zone through them.

Fax: + 55 83 216 7437; e-mail: laqa@quimica.ufpb.br

The drawback is circumvented by using leaping detectors for multi-site detection [10]. After achievement of the analytical signal, the detector is displaced in order to sight another site of the manifold. Washing time is then drastically reduced and sampling rate is improved. Flow systems with leaping detectors [10–13] are very versatile, and permit both serial and parallel monitoring of sample zones. However, the inner volume of the leaping detector plus accessories can be a limiting factor in system design. Before leaping, this volume is filled with the solution related to the first monitoring site, which is inserted into the stream passing through the next monitoring site. In this way, the detector relocation causes an injection that may result in the establishment of undesirable concentration gradients eventually leading to pronounced Schlieren noise [14]. In addition, the detector plus accessories should be washed before the next measurement to avoid carryover effects. These problems are minimized by reducing their inner volume.

LED technology is suitable for downsizing, and the LED/phototransducer sets [14–17] used in flow analysis have been characterized by low inner volumes. In spite of the relatively broad spectral width, LED is overall accepted as a light source in view of its portability, possibility to operate with pulsed current [15], stability, low power dissipation [17], low cost and robustness.

The aim of this work then was to design a LED/phototransistor probe with low inner volume to be used as a leaping detector in a flow-injection system. A bicolour LED (BLED) was used as the source of pulsed radiation in the red and green visible region, and two phototransistors as transducers. The Fe(II)/Fe(III) was chosen as model chemistry to improve the features of the earlier system with multi-site spectrophotometric detection that presents a larger volume of flow cell plus accessories. As an application, speciation of iron in drugs was selected because the drug analysis having iron as a principal component is very important in places where subnutrition causes anaemia.

### Experimental

### *Solutions*

All solutions were prepared with distilled/deionized water and *pro analisi* chemicals.

Fe(II) stock solution (200.0 mg $l^{-1}$ Fe in 0.2 mol $l^{-1}$ HCl) was based on $Fe(NH_4)_2(SO_4)_2.6H_2O$ and prepared as in earlier works [1, 10]. Fe(III) stock solution was similarly prepared but the final volume was completed after drop-wise addition of a 0.002 mol $l^{-1}$ $KMnO_4$ to a constant reddish colour. The Fe(II) stock solution is stable for at least 1 week.

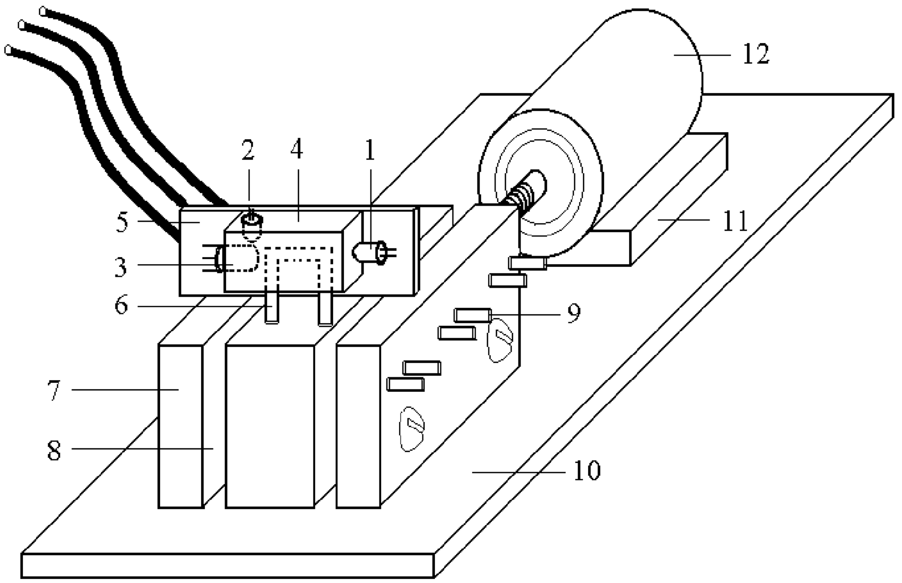

*Figure 1. Schematic presentation of the step-motor driven injector-commutator with the BLED/phototransistor set. 1, BLED; 2, 3, reference and analytical phototransistors; 4, Perspex flow cell; 5, board for the electronic circuit; 6, inlet of the BLED/phototransistor set; 7, Perspex fixed portion of the injector commutator; 8, silicone rubber sheet; 9, outlet to manifold; 10, 11, aluminium bases; 12, step-motor. For details, see text.*

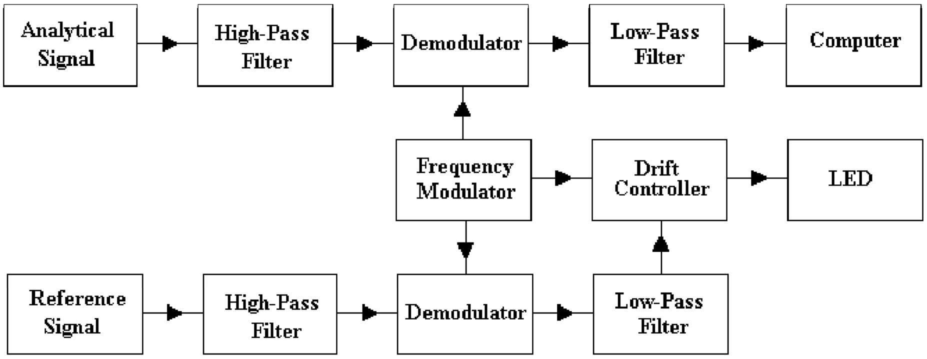

*Figure 2. Block diagram of the BLED/phototransistor set.*

The buffer (0.5 mol $l^{-1}$ acetic acid/ammonium acetate, pH 4.7), the colour (0.25% w/v 1,10 phenanthroline) and the reducing (1.0% w/v ascorbic acid) solutions, $R_1$, $R_2$ and $R_3$ reagents (see figure 4) were prepared as in earlier work [10], the later ones being daily renewed.

Drug samples were purchased in a local pharmacy as syrups and 100-μl aliquots were dissolved in 100 ml of a 0.2 mol $l^{-1}$ HCl solution. Working standards within the 0.0–15.0 mg $l^{-1}$ Fe(II) or Fe(III) range were then daily prepared with this acidity. In order to avoid undesirable concentration gradients, the sample carrier stream was also 0.2 mol $l^{-1}$ in HCl.

### *The flow-injection system*

The system comprised a model IPC-8 Ismatec peristaltic pump provided with Tygon pumping tubes, a manifold build up with 0.8 i.d. Teflon tubing, a home-made photometer similar to that described elsewhere [18], but operating with modulated frequency, a step-motor driven injector-commutator [19] including the BLED/phototransistor set (figure 1), Perspex connectors and accessories. The light source was a BLED, with maximum wavelength and spectral bandwidth of 560 ± 15 nm and 660 ± 10 nm for green and red emission, respectively. The block diagram and electronic circuit of the BLED photometer are presented in figures 2 and 3. The system uses a stabilized source power regulated by 7805 integrated circuit and pulsed currents of 25 and 6 mA for green and red emission, respectively, supplied by the BLED. The pulsed signals from both phototransistors pass through the high-frequency filters in order to minimize low-frequency noise. The filtered signals are sent to demodulating circuits and converted to DC signals by using the control signals from a frequency modulating circuit. The rectified signals are sent to low-frequency filters and the high-frequency noise is minimized. Thereafter, the analytical signal is directly sent as transmittance information, which is converted for

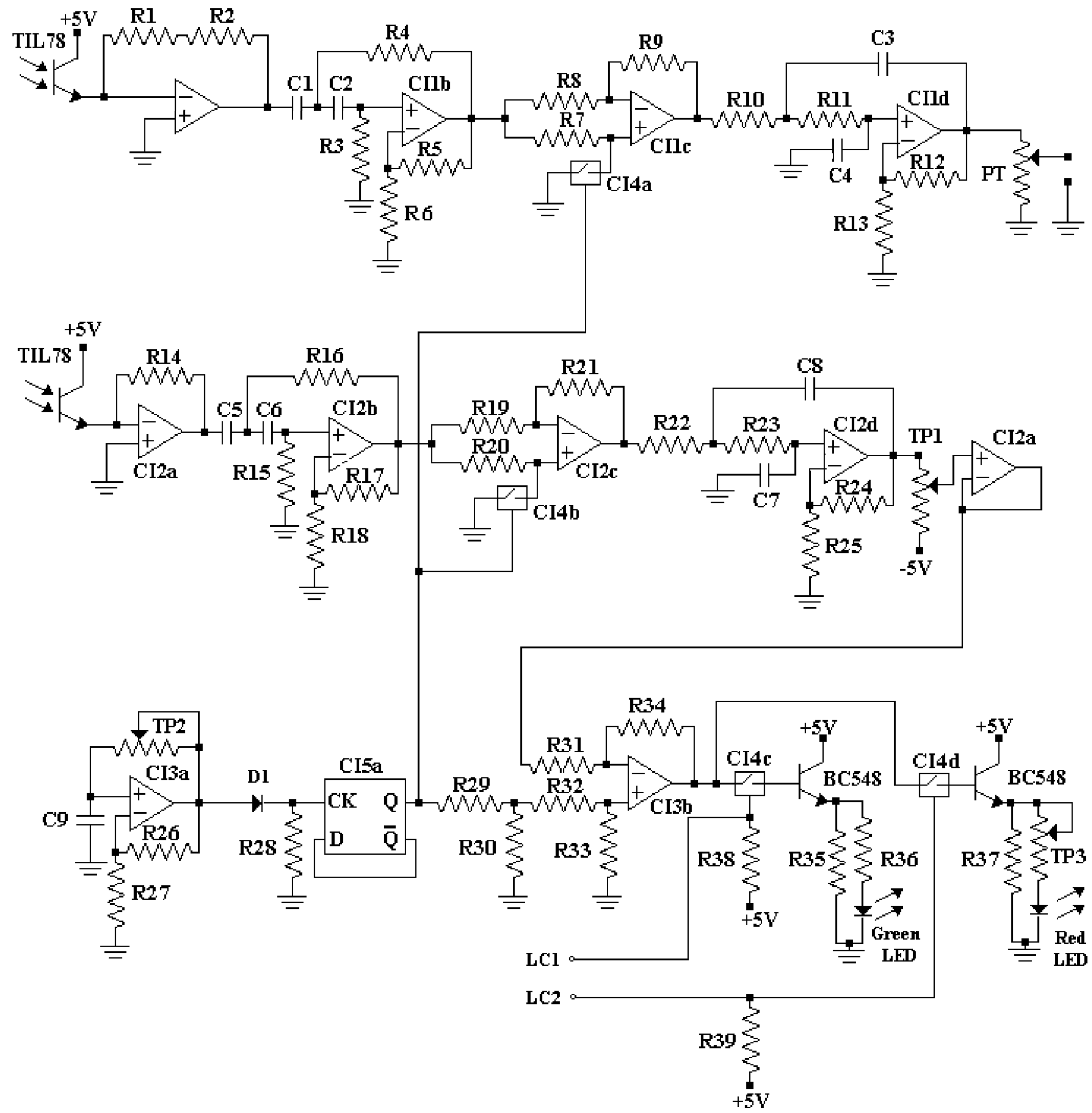

*Figure 3. Electronic circuit of the LED/phototransistor set. TIL78 = phototransistors; R1, R14 = 3.9 MΩ; R2 = 4.7 MΩ; R3–4, R15–16 = 180 kΩ; R5, R6, R12–13, R17–18, R24–27 = 6.8 kΩ; R7–9, R19–21, R28, R30, R38–39 = 10 kΩ; R10–11, R22–23 = 56 kΩ; R29 = 3.3 kΩ, R31–34 = 47 kΩ; R35, R37 = 1 kΩ; R36 = 47 Ω; PT = 10 kΩ; TP1–2 = 47 kΩ; TP3 = 220 Ω; C1–2, C5–6 = 33 nF; C3–4, C7–8 = 330 nF; C9 = 120 nF; D1 = 1N4148 diode; CI1–3 = OP084 quad operational amplifiers; CI4 = CMOS 4066 quad multiplexer; CI5 = CMOS 4013 dual type D flip-flop; CL1–2 = control lines for green and red emissions.*

absorbance by the microcomputer. The reference signal passes through a drift correcting circuit, where it is rearranged in order to provide a controlling signal proportional to the BLED-emitted radiation power. If the radiation power varies, the control signal modifies the amplitude of the square wave tension feeding the radiation source, and the initial emission power is restored.

An IBM PC-compatible microcomputer was used to switch the injector-commutator through the step-motor, to select the wavelength, and for data acquisition and treatment. Software was written in QuickBasic.

The flow diagram of the system with the injector-commutator in the sampling position is shown in figure 4a. The sample was aspirated to fill the sampling loop and its excess was wasted. When the injector-commutator was switched (figure 4b), the sample selected volume was intercalated into its carrier stream, and the established sample zone was further mixed with reagents $R_1$ and $R_2$ permitting the ferroin (1,10-phenanthroline–iron(II) complex) formation inside the coil $B_1$. The processed zone reached the detector and a signal proportional to the iron (II) content in the sample was obtained. The monitored ferroin has a maximum wavelength and spectral bandwidth of $512 \pm 62$ nm. After achievement of peak maximum, the injector-commutator was switched back to the position in figure 4a and the detector was moved downstream. Thereafter, reagent $R_3$ was added allowing iron(III) reduction and additional ferroin formation inside coil $B_2$. The coloured sample was monitored again, yielding a signal now proportional to the total iron content in the sample.

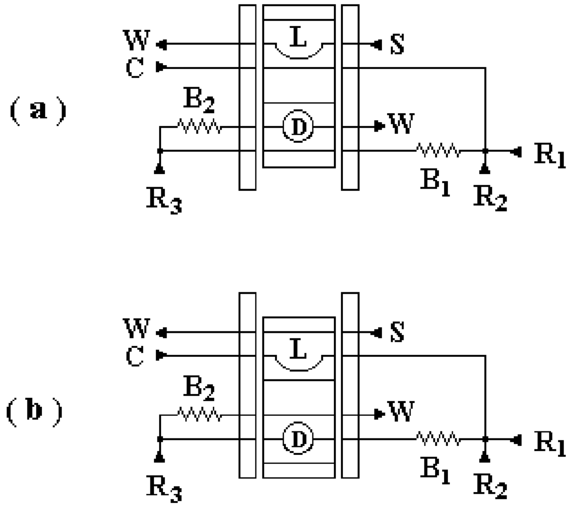

*Figure 4. Flow diagram of the proposed system with the injector-commutator in the sampling (a) and injection (b) position. S, sample aspirated at 2.0 ml min$^{-1}$; L, 20-cm sampling loop (ca. 100 μl); C, sample carrier stream at 4.0 ml min$^{-1}$; $R_1$, $R_2$, $R_3$, buffer, colour and reducing reagents at 1.0 ml min$^{-1}$; $B_1$, $B_2$, 100-cm reaction coils; D, leaping detector; W, waste. For details, see text.*

*Procedure*

Initially, a procedure similar to that suggested by Karlberg and Pacey [20] was carried out to estimate the inner volume of the leaping detector plus accessories. The detector plus its inlet and outlet connections acted as a sampling loop which inserted a 1000.0 mg l$^{-1}$ Fe(II) solution into the system of figure 4. The commutator was moved only once, and the detector remained in the first monitoring site. The solution leaving the flow system was collected into a 10-ml volumetric flask. The volume of the flask completed with the stream leaving the flow system, its absorbance measured in a HP 8453 Hewlett-Packard spectrophotometer and compared with that related to a 10.0 mg l$^{-1}$ Fe(II) solution, allowing the inner volume of the detector plus accessories to be calculated.

After evaluating other characteristics of the detector, e.g. optical path, illuminated volume, stability, response time and linearity, the system shown in figure 4 was applied to iron speciation in drugs. Precision of the results was evaluated as the relative standard deviation estimated after eightfold processing of typical samples (4.0–15.0 mg l$^{-1}$ Fe (II) or total iron) and accuracy was assessed by running some already analysed [21] samples.

**Results and discussion**

The optical path of the leaping detector was measured as 8.0 mm. Because the tubular flow cell had an inner diameter of 2.6 mm, the illuminated volume was calculated as 42 μl. With this volume, signal-to-noise ratio was suitable and a baseline noise < 0.002 A was observed for the system in figure 4.

The total inner volume of the leaping detector corresponding to the illuminated volume plus the inlet and outlet connections plus the inner volume of the injector-commutator was determined [20] as 64 μl. Limitations due to this inner volume are usually more severe when serial monitoring is concerned because the solution inside this volume is transferred to the other monitoring site and the remaining sample zone is depleted. This aspect is less relevant in parallel monitoring where another sample zone is monitored at the next site. Although the present system involved serial monitoring, the volume of the detector plus accessories did not impair its performance and Schlieren noise was not observed even immediately after detector leaping from sites with different ion concentrations. A comparison between illuminated and total inner volumes reveals that only ~11 μl act as dead volumes at both sides of the illuminated region of the leaping detector. It should be stressed that the total inner volume of the leaping detector was only ~16% relative to that reported for an analogous system [10].

The detector response time was suitable for the proposed flow-injection procedure. In fact, after stopping the peristaltic pump when top peak was attained, a steady state absorbance was recorded, indicating that both chemical reaction and measurement were completed. Moreover, the time for baseline restoration after detector leaping was always < 1 s and depended on the flow cell washing time rather than the detector response time.

Thermal drift was observed mainly for the green BLED (~0.005% min$^{1}$), whereas a stable situation was noted for the red BLED. As the proposed procedure used the green BLED, it was decided to reduce the dissipated power and to exploit double detection. The former improvement was attained by using a pulsed current (310 Hz—symmetrical square wave) and the later by using two phototransistors in order to attain a situation analogous to that found in double-beam spectrophotometry. Thermal drift was then completely circumvented.

Parallel experiments involving dye solutions (copper sulphate or potassium permanganate) confirmed that the detector presented a linear dynamic range for absorbance + concentration for these solutions. For the pro-

*Table 1. Statistical parameters of linear regression for Cu(II), $KMnO_4$ and ferroin analytical curves.*

| | |
|---|---|
| Cu(II) | $A = 0.0032 + 2.8$ [Cu]; $r^2 = 0.9999$ ($n = 5$)<br>Linear range: 0.020–0.20 mol l$^{-1}$ |
| $KMnO_4$ | $A = 0.0023 + 1.1$ [$KMnO_4$]; $r^2 = 0.9998$ ($n = 6$)<br>Linear range: 0.020–0.20 mmol l$^{-1}$ |
| Ferroin (sampling position) | $A = 0.0073 + 0.0094$ [Fe]; $r^2 = 0.9990$ ($n = 5$)<br>Linear range: 1.0–20.0 mg l$^{-1}$ |
| Ferroin (injection position) | $A = 0.0040 + 0.0071$ [Fe]; $r^2 = 0.9996$ ($n = 5$)<br>Linear range: 1.0–20.0 mg l$^{-1}$ |

*Table 2. Iron speciation in synthetic samples as determined by the proposed system and by a conventional procedure [21].*

| | Expected value | | | Determined value | | |
|---|---|---|---|---|---|---|
| *Sample* | *Fe(II)* | *Fe(III)* | *Total Fe* | *Fe(II)* | *Fe(III)* | *Total Fe* |
| 1 | 1.6 | 2.4 | 4.0 | 1.5 (±2.0%) | 2.3 (±2.6%) | 3.8 (±1.7%) |
| 2 | 2.4 | 1.6 | 4.0 | 2.3 (±1.9%) | 1.7 (±2.2%) | 4.0 (±1.2%) |
| 3 | 3.2 | 2.4 | 5.6 | 3.1 (±1.3%) | 2.4 (±1.8%) | 5.5 (±1.3%) |
| 4 | 3.2 | 4.0 | 7.2 | 3.2 (±1.7%) | 4.0 (±2.5%) | 7.2 (±1.8%) |
| 5 | 4.0 | 3.2 | 7.2 | 3.7 (±2.0%) | 3.4 (±2.2%) | 7.1 (±1.0%) |
| 6 | 2.4 | 4.6 | 7.0 | 2.5 (±1.0%) | 4.7 (±1.8%) | 7.2 (±1.5%) |

Results are in mg $l^{-1}$ based on eight replications.
The values in parentheses are the relative standard deviation ($n = 8$).

posed system, linearity was verified by injecting Fe(II) standard solutions and measurements were carried out at the two monitoring sites. The relationship between peak height and analyte concentration was linear up to ~20 mg $l^{-1}$ Fe, which corresponded to an absorbance value of only ~0.22 AU. This low absorbance value is a consequence of the low overlap between emission and absorption spectra. A similar effect was reported by Trojanowicz and Szpunar–Lobinska [16] who reported that only 21% of the LED emission spectrum could be absorbed by ferroin. Although sensitivity was reduced, the applicability of the method was not impaired.

Figure 5 refers to successive injections of a 10.0 mg $l^{-1}$ Fe(II) with measurements related to different leaping times, $t_l$. For $t_l < 5$ s, no signal was recorded at the first monitoring site because the sample zone did not reach the detector before its leaping, and the entire zone was monitored only at the second site. For $5 < t_l < 11$, the frontal portion of the sample zone was quantified at the first monitoring site and slices were transferred to the second site. For $t_l = 11$ s, a slice at the most concentrated portion of the sample zone was transferred and maximum depletion of the sample zone was then verified at the second site. For $11 < t_l < 16$ s, suitable measurements could be performed in both monitoring sites. For $t_l > 16$ s, detector leaping towards the second monitoring site occurred too late, and the central portion of the sample zone passed through the second monitoring site before arrival of the detector, thus limiting sensitivity at this site. Leaping instants were then selected, as 16 and 27 s. Sampling throughput was then 133 $h^{-1}$ (266 measurements) which means 1.1 µg 1,10 phenanthroline consumed per sample. These figures are much better than those earlier reported [10].

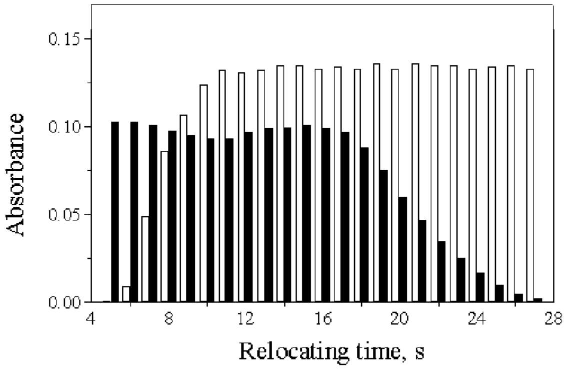

*Figure 5. Influence of relocating time $t_l$. The figure refers to 10.0 mg $l^{-1}$ Fe(II) introduced into the system of figure 4. Each white and black bar is the absorbance measurements (rsd = ±1.5%, n = 6) in the injection and sampling positions. For details, see text.*

The proposed system is very stable, and no baseline drift is noted after 8-h operation periods. Table 1 refers to its application to synthetic samples, and reveals the agreement between results obtained with the proposed system and expected values. Mean deviations were usually ~±4.2% for Fe(II) or ±5.2% for Fe(III).

After applying the system to drug speciation, precise results were obtained, and the overall relative standard deviation was estimated as 1.6% after eightfold successive processing of typical samples with 5.0–15.0 mg $l^{-1}$ Fe(II) in the injectate. In the assayed samples, iron was present as the ferrous ion, but for some samples, the total iron content was higher then the Fe(II) content, in view of drug or solution oxidation. Agreement between results obtained with the proposed and reference [21] procedures was confirmed by applying the paired *t*-test, and no statistic differences between results at the 95% probability level were found.

### Conclusions

Performance of the proposed system is superior relative to the earlier system with similar dimensions in terms of sampling rate, reagent consumption, susceptibility to contamination and portability. This last feature makes it suitable to be used in routine analysis in places where drugs are handled. It provides iron speciation and, for drugs based on ferrous sulphate, it may forward additional information about drug oxidation.

The designed leaping detector causes the exchange of very small liquid aliquots between different monitoring sites. Schlieren noise is then minimized and inter-contamination reduced. The presence of ascorbic acid inside the detector immediately after its leaping towards the first monitoring site does not alter results for Fe(II).

For other applications more susceptible to Schlieren effects, the detector can be used in the dual-wavelength mode, and preliminary experiments pointed out this potentiality. For sensitivity enhancement, use of others reagents [16] or exploitation of spectrum shifters is recommended. Studies focusing on these points are presently in progress.

### Acknowledgment

This work was partially supported by a consortium project involving CNPq (Brazil) and JNICT (Portugal). CNPq and CAPES grants to M.C.U.A., R.S.H. and S.R.B.S. are appreciated.

### References

1. Hansen, E. H., Muller, H. and Muller, V., 1990, *Analytica Chimica Acta*, **230**, 113–123.
2. Stewart, J. W. B. and Ruzicka, J., 1976 *Analytica Chimica Acta*, **82**, 137–144.
3. Ramasamy, S. M. and Mottola, H. A., 1981, *Analytica Chimica Acta*, **127**, 39–46.
4. Araújo, A. N., Lima, J. L. F. C., Rangel, A. O. S. S., Alonso, J., Bartroli, J. and Barber, R., 1989, *The Analyst*, **114**, 1465–1468.
5. Valcárcel, M., Luque de Castro, M. D. and Fernandes, A., 1987, *Analytica Chimica Acta*, **193**, 107–118.
6. Ruzicka, J. and Hansen, E. H., 1988, *Analytica Chimica Acta*, **114**, 1–27.
7. Toei, J., 1988, *The Analyst*, **113**, 475–478.
8. Dahl, J. H., Espersen, D. and Jensen, A., 1979, *Analytica Chimica Acta*, **105**, 327–333.
9. Valcárcel, M., Luque de Castro, M. D., Lazaro, F. and Rios, A., 1989, *Analytica Chimica Acta*, **216**, 275–288.
10. Zagatto, E. A. G., Bergamin F$^{o}$, H., Brienza, S. M. B., Arruda, M. A. Z., Nogueira, A. R. A. and Lima, J. L. F. C., 1992, *Analytica Chimica Acta*, **261**, 59–65.
11. Nogueira, A. R. A, Brienza, S. M. B., Zagatto, E. A. G., Lima, J. L. F. C. and Araújo, A. N., 1993, *Analytica Chimica Acta*, **276**, 121–125.
12. Gomes N$^{o}$, J. A., Nogueira, A. R. A., Bergamin F$^{o}$, H., Zagatto, E. A. G., Lima, J. L. F. C. and Montenegro, M. C. B. S. M., 1994, *Analytica Chimica Acta*, **285**, 293–299.
13. Nogueira, A. R. A, Brienza, S. M. B., Zagatto, E. A. G., Lima, J. L. F. C. and Araújo, A. N., 1996, *Journal of Agricultural and Food Chemistry*, **44**, 165–169.
14. Betteridge, D., Dagless, E. L., Fields, B. and Graves, N. F., 1978, *The Analyst*, **103**, 897–908.
15. Hooley, D. J. and Dessy, R. E., 1983, *Analytical Chemistry*, **55**, 313–320.
16. Trojanowicz, M. and Szpunar-Lobinska, J., 1990, *Analytica Chimica Acta*, **230**, 125–130.
17. Dasgupta, P. K., Bellamy, H. S., Liu, H., Lopes, J. L., Loree, E. L., Morris, K., Petersen, K. and Mir, K. A., 1993, *Talanta*, **40**, 53–74.
18. Araújo, M. C. U., Santos, S. R. B., Silva, E. A., Veras, G., Lima, J. L. F. C. and Lapa, R. A. S., 1997, *Quimica Nova*, **20**, 137–145.
19. Pasquini, C. and Raimundo, I. M., Jr, 1997, *The Analyst*, **122**, 1039–1044.
20. Karlberg, B. and Pacey, G. E., 1989, *Flow Injection Analysis. A Practical Guide* (New York: Elsevier Science).
21. Jeffery, G. H., Basset, J., Mendham, J. and Denney, R. C., 1989, *Vogel's Textbook of Quantitative Chemical Analysis*, 5th edn (Woolwich, UK: Longman Scientific & Technical).

